# The Influence of Fluidized Bed Combustion Fly Ash on the Phase Composition and Microstructure of Cement Paste

**DOI:** 10.3390/ma12172838

**Published:** 2019-09-03

**Authors:** Michał A. Glinicki, Daria Jóźwiak-Niedźwiedzka, Mariusz Dąbrowski

**Affiliations:** Institute of Fundamental Technological Research, Polish Academy of Sciences, Pawińskiego 5b, 02-106 Warsaw, Poland

**Keywords:** clean coal combustion, fluidized bed fly ash, microstructure, phase composition, portlandite, unburned carbon

## Abstract

Fly ashes from coal combustion in circulating fluidized bed boilers in three power plants were tested as a potential additive to cement binder in concrete. The phase composition and microstructure of cement pastes containing fluidized bed fly ash was studied. The fractions of cement substitution with fluidized bed fly ash were 20% and 30% by weight. X-ray diffraction (XRD) tests and thermal analyses (derivative thermogravimetry (DTG), differential thermal analysis (DTA) and thermogravimetry (TG)) were performed on ash specimens and on hardened cement paste specimens matured in water for up to 400 days. Quantitative evaluation of the phase composition as a function of fluidized bed fly ash content revealed significant changes in portlandite content and only moderate changes in the content of ettringite.

## 1. Introduction

Constantly evolving technologies of coal combustion, with the aim of improving energy efficiency, include among others the more efficient fluidized bed combustion (FBC) systems. A well designed boiler can combust coal with relatively high efficiency and an acceptable level of gas emissions. FBC boilers operate at lower temperatures (800–900 °C) compared to conventional pulverized coal combustion (1400–1700 °C) [1]. The diversity of work conditions of increasingly used fluidized bed boilers makes it possible to use them to burn high-sulfur fuels [2]. The fuel flexibility is an important characteristic of FBC boilers [3]. The best example is circulating fluidized bed combustion (CFBC), which provides low emissions of SO_2_ and NO_x_ to the atmosphere [3,4,5] while maintaining the efficiency of coal combustion. 

A systematic increase of coal combustion by-products from fluidized boilers has been observed in numerous countries. One of the ways of managing CFBC by-products is to use them as an additive to cement and concrete. Studies on the possibility of the use of CFBC fly ash as a cement or concrete additive have been carried out for several years [1,6,7,8,9,10,11,12], but still there are no normative regulations regarding the use of this kind of fly ash as a cement additive. 

Some researchers have performed investigations on the material characteristics and mechanical properties of CFBC fly ash in cement-based composites. It has been shown that CFBC fly ash is characterized by high variability of chemical and phase composition [13]. The dominant CFBC particles were coarse and had an irregular flaky shape characterized by a broad particle size range [12] which resulted in high demand of water and admixture [14]. The high content of anhydrite in CFBC resulted in excessive expansion in cement matrix composites due to abundant ettringite formations [15]. Moreover, most of the CFBC fly ash had less pozzolanic activity than the conventional siliceous fly ash [7] and some of the CFBC fly ashes do not meet the standard requirements to be classified as either Class C or Class F fly ash [11]. The main reason for limiting the use of CFBC fly ash in blended cements is a high content of sulfates (mostly anhydrite), and free calcium oxide, whose presence contribute to creation of secondary ettringite—negative from point of view of the durability of cement matrix composites [16]. The overview of the effect of reactive mineral additives on the microstructure and phase composition of the cement hydration products was presented in [17]. Rajczyk et al. [18] used the DTA method to follow the hydration process of cement containing fluidized bed combustion fly ash. Based on endothermic peaks attributed to the dehydration of phases formed on hydration, the conditions leading to the formation of so-called delayed ettringite were found. Gazdiča et al. [1], based on the progress of hydration of blended cements, proved that ettringite formed from anhydrite contained in fluidized bed ash was one of the hydration products built during the formation of the cement stone structure, thus ettringite was not the cause of negative volume changes.

However, despite these observations, it has also been shown that application of CFBC fly ash simultaneously with siliceous fly ash or slag as a binder component makes it possible to obtain a durable cement-based composite. Nguyen et al. [19] used CFBC fly ash as a sulfate activator which significantly improved the mechanical properties of the modified high-volume fly ash cement pastes at early ages. The accelerated hydration of C_3_S and more precipitated ettringite formation of hardened pastes confirmed the important role of CFBC fly ash to enhance the mechanical properties of the cement pastes. Hlaváček et al. [20] used CFBC fly ash in a ternary binder (plus siliceous fly ash and calcium hydroxide) and they obtained a compressive strength of paste equal to 32 MPa after 28 days of curing. The ternary hydraulic binder containing CFBC fly ash was also analyzed by Škvára et al. [21]. They stated that a ternary binder possessed strength values comparable to those of Portland cement. Lin et al. [22] investigated the properties of controlled low strength material with circulating fluidized bed combustion ash and recycled aggregates. They showed that CFBC hydrated ash resulted in a higher compressive strength when compared with desulfurized slag, but had a lower compressive strength than coal bottom ash. Chi [23] characterized the mortars with CFBC fly ash and ground granulated blast-furnace slag and he found that this kind of fly ash had the potential to partially replace the cementing materials, but the proportion of CFBC fly ash was recommended to be limited to a maximum of 20% due to the decreasing of compressive strength and the increasing of initial setting time of mortars. Dung et al. [24] analyzed the application of raw circulating fluidized bed combustion fly ash and slag as eco-binders for a novel concrete without ordinary Portland cement. They found that this new binder behaves similar to ordinary Portland cement. The pastes showed good workability, proper setting times, and sufficient compressive and tensile strength, which were improved with the decrease of water/binder (w/b) ratio and with the increase of hydration time. Chen et al. [25] tested expansion properties of cement paste with circulating fluidized bed fly ash. They stated that the curing method is of great importance. The expansion development in its early days was dominated by the ettringite, and its quantity and morphology were seriously affected by the inadequateness of water. The application of CFBC fly ash improved concrete durability. Jóźwiak-Niedźwiedzka [26] analyzed the influence of CFBC fly ash on the chloride and scaling resistance of concrete. She showed that the cement replacement by 15% and 30% CFBC fly ash provided higher chloride resistance, but only replacement by 15% CFBC fly ash can be used to achieve concrete scaling durability. Kubissa et al. [27] showed that use of 25 wt % fluidized fly ash as cement replacement improved concrete water permeability and sorptivity, as well as chloride resistance. On the other hand, Czarnecki et al. [28] found that the depth of concrete carbonation increased with increasing of the content of CFBC fly ash but at the same time they obtained similar results for ordinary siliceous fly ash. 

Juenger et al. [29] have recently presented a review paper on emerging supplementary cementitious materials (SCM). They concluded that with the increase in demand for conventional SCM and the simultaneous reduction in supply, there is a great need to find new sources of materials that provide comparable or better properties to highly used fly ash and slag sources. So, the research into new sources of SCMs (like fly ash from bed fluidized coal combustion that does not meet current specifications) and their impact on cement matrix properties has been increasing in recent years, and has great likelihood to further increase as the demand for these materials grows.

Despite many publications on the possible application of the CFBC fly ash in concrete technology, the problem of its proper use remains unresolved. An increasingly technical rigorous regime during the combustion of coal in fluidized bed boilers has contributed to improving the stability of the composition and reducing the amount of undesirable ingredients. Research on the new kinds of fly ash from circulating fluidized bed combustion has considered their specific characteristics of phase composition and paste microstructure. This paper aims to show some relationships between the differences in mineral composition and origin of burning coal. The phase composition as well as microstructure of cement pastes with various content of CFBC fly ash have been studied. A prolonged time of curing has been applied to determined how the replacement of Portland cement by CFBC fly ash would change the microstructure and phase composition of paste stored in high-humidity conditions.

## 2. Experimental Program

### 2.1. Materials and Methods 

The fly ash used in the research came from the circulating fluidized bed combustion process in three Polish power stations: Katowice (K), Siersza (S) and Turów (T). Ashes were formed from the combustion of hard coal (K, S) and lignite (T). CFBC fly ash chemical analysis was carried out in accordance with European standards and is presented in Table 1. 

Comparing the chemical composition to the requirements of PN EN 450-1, an excess of SO_3_ caused by anhydrite in the fly ash is shown. Ordinary Portland cement 32.5 R from “Małogoszcz” cement plant was used. The composition and physical properties are given in Table 2. Binders of the above ingredients were prepared by replacing 20% and 30% of the cement mass by fluidized fly ash K, S or T. For example, T20 corresponds to 20% fly ash from Turów (lignite burning), and K30 corresponds to 30% fly ash from Katowice (hard coal burning). The detailed composition of pastes is presented in Table 3. The difference in density between fly ash and cement was neglected. The cement paste with a constant water to binder ratio 0.5 was formed using prisms with dimensions 40 × 40 × 160 mm. Storage of the molds took place for 48 hours in a chamber with a humidity of 95%; after this period of time the specimens were kept in water and matured for 400 days at 20 ± 2 °C.

### 2.2. Test Methods

The CFBC fly ash was analyzed at macro and micro levels. For the analysis of mineral composition X-ray (XRD) (TUR-M62/VEB TuR Dresden, Germany) and thermal analysis (DTA, TG and DTG) (SDTQ 600, TA Instruments, Artisan Technology Group, Champaign, USA) were used. Tests were carried out with powder specimens which were also divided by fraction: above and below 0.045 mm, separated by measuring the fineness of ash in accordance with PN-EN 451-2. The content of free lime CaO_free_ was measured according to standard glycol method, PN-EN 451-1. After the chemical and physical characterization of CFBC fly ash, the samples proceeded to the second part of the research aimed to determine the phase composition of cement paste. The cement–fly ash binder was investigated by thermal analysis and X-ray diffraction, as well as microscopic observation after 28, 200 and 400 days of maturity.

Macroscopic analysis was performed with the naked eye. In order to identify differences in behaviour and colour of specimens, they were tested by reaction with an aqueous solution of HCl (1:3) and phenolphthalein (1% in ethanol), respectively. Determination of color specimens was carried out by applying a sheet of colours recommended by PN-EN 12407.

The flexural strength testing was carried out after 28 days of maturity, on three 40 × 40 × 160 mm beams for each paste composition according to the three-point bending test presented in the Standard PN-EN 191-1, [30]. The test was carried out using the Lloyd Instruments testing machine, type EZ50 (Lloyd Instruments LTD, Hampshire, UK). Compressive strength of analyzed pastes was determined on halves of beams broken during bending test (after 28 days of curing). The test was performed using appropriate inserts, enabling measurements in accordance with PN-EN 196-1.

The microstructure of the powder and paste specimens was analyzed using scanning electron microscope (SEM) LEO 1530 (Carl Zeiss Microscopy GmbH, Jena, Germany). The surface of specimen observation in SEM was not less than 1.0 cm^2^ over the range of magnifications from 200× to 50,000×. The specimens were coated with a layer of carbon with a thickness of about 10 nm using the device BalTec SCD 005, CEA 035 adapter (BAL-TEC GmbH, Schalksmühle, Germany). Thermal analysis was performed at the following setup: heat rate 10 °C/min in platinum crucibles for the standard sample of Al_2_O_3_ in the air atmosphere. X-ray diffraction analysis was performed using diffractometer TUR-M62 (TUR-M62/VEB TuR Dresden, Germany), with the following conditions: CuK_α_ radiation, monochromator filter, 40 kVA voltage and 20 mA current of the X-ray lamp, BDS-7 meter, 0.05 s step, 5 s time constant.

## 3. Test Results and Discussion

Results of macroscopic analysis are presented in Table 4. The most important observations made in a part of this study was the colour test variation from brownish-grey (5YR4/1) for CFBC fly ash from Siersza to light olive-grey (5Y6) for CFBC fly ash from Turów. It is probably related to the amount of unburned coal in the analyzed specimen and the type of burned material: Siersza (hard coal) and Turów (lignite). The darker colour corresponds to the higher coal content, which is also visible on the basis of LOI results in the analyzed fly ashes.

Reaction with HCl, for each of the fly ashes passed with the same intensity of the characteristic light “effervescence”. So it should therefore be expected these specimens had similar content of CaO_free_. Higher fineness (36.1%) was exhibited by CFBC fly ash (T); it was several age points higher than the other tested fly ashes (K and S).

**Figure 1:**
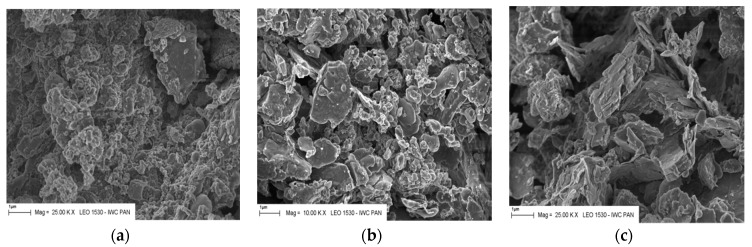
Microstructure of CFBC ashes from different power stations: (**a**) T, lignite burning; (**b**) S, hard coal burning; and (**c**) K, hard coal burning. Magnification 25,000×, scale bar = 1 µm.

**Figure 2:**
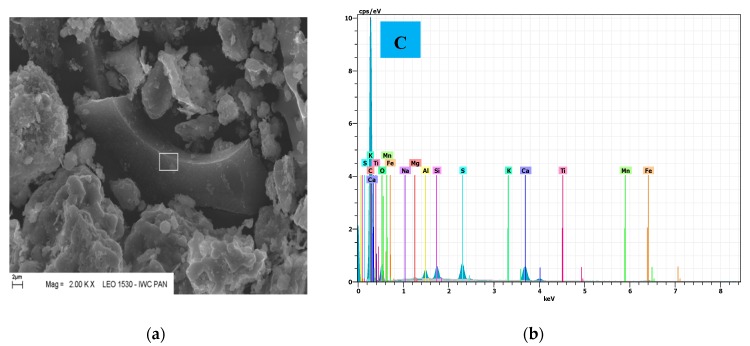
Microstructure of CFBC fly ash grain with visible unburned coal particle (**a**) and analysis in microarea (**b**); magnification 20,000×, scale bar = 2 µm.

Observations in the scanning electron microscope showed typical grains corresponding to fly ash from fluidized bed combustion boilers. In Figure 1 a high surface development and irregular shape of the fly ash particles is visible. The largest observed particle size reached 120 μm for T fly ash and 80 μm for K and S fly ash. Also, relics of coal (often like char, which was verified by EDS analysis) are shown, which are disadvantageous from the standpoint of application of these fly ashes in cement (Figure 2). The non-hydroxylated clay minerals and combustion sorbent calcium carbonate have been found.

The results of flexural strength and compressive strength of tested pastes are shown in Table 5. The results of flexural strength for all pastes with CFBC were in the range of 5.2–6.4 MPa and differences were statistically negligible. The reference paste revealed a lower value of flexural strength (4.6 MPa). A much higher (about 50%) 28-day compressive strength of pastes made with the 20% replacement of cement by CFBC fly ash from hard coal burning (K and S) was observed compared to the CEM I reference paste. Specimens with CFBC fly ash from lignite burning (T) achieved slightly lower values of compressive strength compared to specimens with fly ash from hard coal burning. The increase in CFBC fly ash content influenced the decrease of compressive strength of pastes. This tendency is much more visible with increase of CFBC addition to 30%. The obtained results are consistent with the literature data [31,32]. Šiler et al. [31] showed that the replacement of cement by 10, 20 and 40 wt % of fluidized bed combustion fly ash influenced on the increase of compressive strength compared to ordinary cement paste in the early age of hydration (up to 28 days). Hanisková et al. [32] analyzed the influence of fly ash from fluidized bed combustion on mechanical properties of pastes. They showed that the highest values for 28-day compressive strength were achieved by pastes with 30–40% replacement of cement by CFBC fly ash, twice as much as the reference paste without fly ash.

The phase composition was evaluated using the thermal analysis method. The loss on ignition and weight loss of analyzed specimens were associated with the relics of clay minerals (temperature up to 350 °C) as well as the oxidation of unburned coal residue, and they were used to determine the content of Ca(OH)_2_ and CaCO_3_. The total loss on ignition was identified by heating fly ash to a temperature of 1000 °C. The results of thermal analysis are presented in Table 6. The obtained measurements show that a total loss on ignition depends not only on the loss of the unburned coal relics, but also the distribution of carbonates, portlandite and disposal of residual clay mineral water. Therefore, the LOI determined according to PN-EN 450-1 does not clearly reflect a precise content of unburned coal. This phenomena is the most visible for CFBC fly ash from Turów power plant (lignite burning), where the presence of the LOI (at 1000 °C) is mainly due to the calcium carbonate content (4.80%), and the relics of unburned coal accounted for only a fraction of a percent in the tested ash (0.8%).

**Figure 3:**
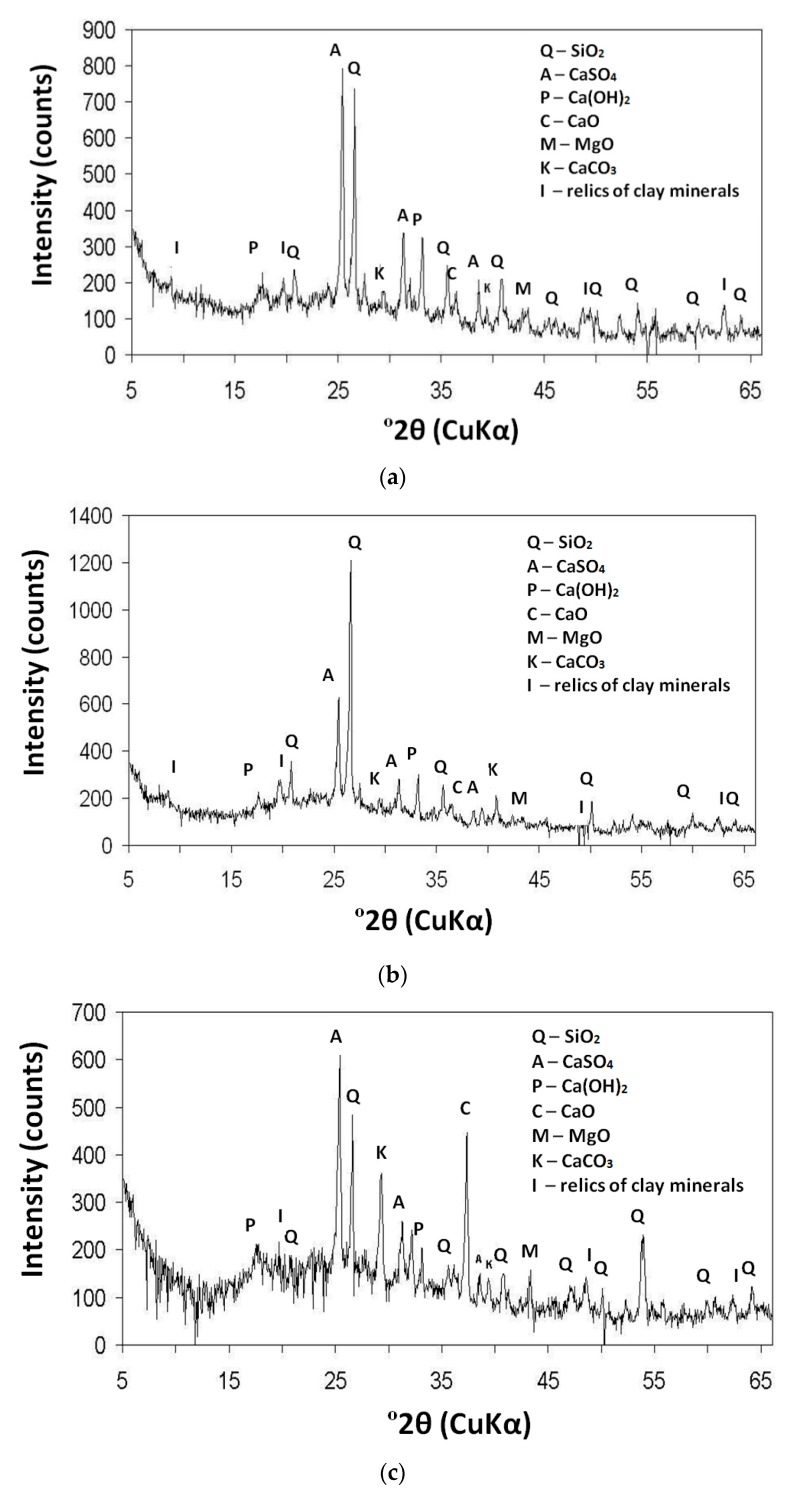
X-ray (XRD) patterns of CFBC fly ash from (**a**) S (hard coal); (**b**) K (hard coal); and (**c**) T (lignite).

The X-ray analysis performed on whole fraction of fly ash revealed the presence of crystalline phases such as anhydrite II (CaSO_4_), portlandite (Ca(OH)_2_), quartz (SiO_2_) and small quantities of clay minerals and calcium (CaO) and magnesium (MgO) oxide. A clear difference between the CFBC fly ash from the hard coal (K and S) and lignite (T) combustion is visible. There is much more portlandite (Ca(OH)_2_) and calcite (CaCO_3_) in the fly ash from the combustion of lignite (fly ash T) compared to hard coal combustion. Diffraction patterns of the analyzed specimens are presented in Figure 3.

For the further study two CFBC fly ashes were selected: K and T as representatives of the waste materials from the fluidized bed combustion of hard coal and lignite, respectively, with a low content of SO_3_. The analysis of the phase composition of cement pastes with 20% and 30% replacement of the cement mass by CFBC fly ash after 28, 200 and 400 days of maturation in water was conducted. 

Based on the results of thermal analysis of cement paste, the chemical-bound water content in hydration products (HI) was determined. 

**Figure 4:**
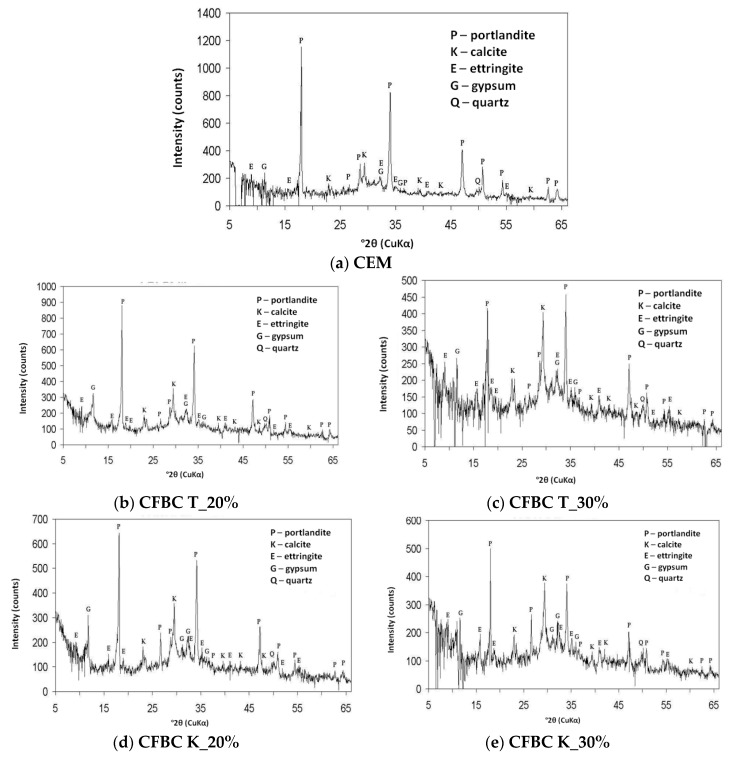
XRD patterns of cement pastes matured in water for 400 days. (**a**) Without any addition; (**b**) with 20% CFBC fly ash T; (**c**) with 30% CFBC fly ash T; (**d**) with 20% CFBC fly ash K; (**e**) with 30% CFBC fly ash K.

The volume of HI is considered as the loss of water at temperatures up to 400 °C, which is associated with hydrated calcium silicates (C–S–H) and calcium aluminosulfate. Additionally, the presence of calcium carbonate (CC) was detected in the hardened cement paste, and it was considered as the mass loss at 800–1000 °C. XRD analysis of the above hydration products revealed that small amounts of ettringite and calcium sulfates mainly in the form of gypsum were present. Diffraction patterns of the cement paste specimens are shown in Figure 4. After the qualitative analysis in order to present the quantitative changes occurring during the hydration process, the change index (WZ) was introduced, which is used as standard procedure [33]. The above method was described in [12].

The XRD pattern revealed portlandite as a major phase in the reference cement paste specimen analyzed after 400 days curing in water. The products of the cement hydration in the forms of portlandite, gypsum and ettringite, and products of the carbonation of the hydrated cement in the form of calcite were found in the specimens made with addition of the CFBC fly ash. The content of calcite was increased with increasing fly ash content. 

Value of change index WZ was defined as the ratio of selected characteristic of the tested cement paste with CFBC fly ash to the same characteristic of the reference cement paste, expressed as a percentage. As a reference, a cement paste without addition of CFBC fly ash matured in the same period of time as specimens with addition of fly ash was chosen. The results of the comparison of phase changes in the tested cement pastes are presented in Table 7. Due to the increased sulfate and calcium ion content in the analyzed fly ashes, the most relevant in the examination of cement paste are ettringite content and amount of calcium carbonate introduced. It was revealed that the increase of ettringite and calcium carbonate content in the hardened cement paste was related to the increase in CFBC fly ash content.

It was noted that the specimens without the addition of CFBC fly ash (i.e., reference specimens) had less than 15% and 13% ettringite content after 400 days of maturing than specimens with 30% fly ash, T30 and K30. respectively. It was surprising that the largest difference in ettringite content between reference specimens and those with addition of CFBC fly ash was visible for the first 28 days of the maturation period. Ettringite decline in subsequent periods is probably associated with the progress of hydration and transformation of ettringite in monosulfate or connected the SO_4_^2–^ ions to the rising C–S–H phase with a low C/S ratio. Hydrated calcium silicates formed by the pozzolanic reaction decrease the content of the portlandite in the cement paste. This is consistent with the results regarding siliceous fly ash. It is known that the addition of the siliceous fly ash to Portland cement generally reduces the amount of portlandite and this is often accompanied with an increase in the amount of C–S–H with reduced Ca/Si ratio and AFm phases due to a higher content of Al_2_O_3_ in fly ash. The AFm phase of Portland cement refers to a family of hydrated calcium aluminates based on the hydrocalumite-like structure of 4CaO·Al_2_O_3_·13–19H_2_O, [34]. Also, the content of ettringite varies depending on the reactivity of the siliceous fly ash used. Studies to characterize the microstructure of concrete modified with addition of calcareous fly ash were performed by Glinicki et al. [35]. They showed that the addition of calcareous fly ash reduced the content of portlandite in the matrix by 45–74%. Results of tests conducted by Tishmack et al. [36] showed that the products of cement hydration incorporating calcareous fly ash included lower amounts of ettringite and higher content of AFm phases, including mainly monosulfates.

**Figure 5:**
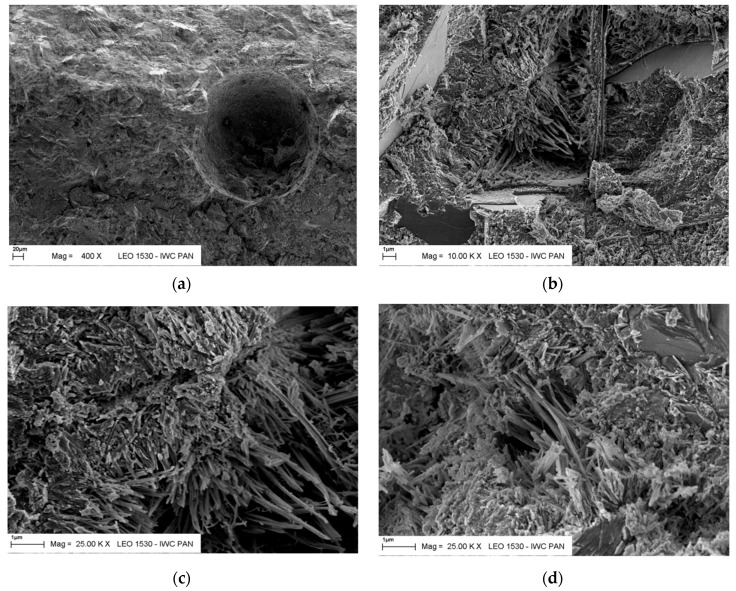
Microphotographs of hardened cement paste microstructure without CFBC fly ash addition, matured in water for 400 days. (**a**) Empty air void without crystalline hydration products; (**b**) C–S–H, ettringite, relics of clinker; (**c**) C–S–H and cluster of elongated ettringite needles; (**d**) C–S–H, ettringite, relics of clinker.

**Figure 6:**
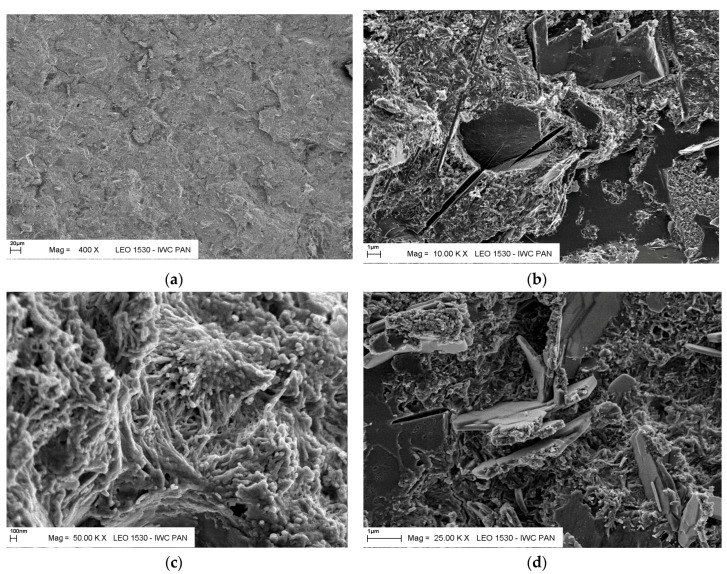
Microphotographs of hardened cement paste with CFBC fly ash (K30), matured in water for 400 days. (**a**) Cement paste; (**b**) C–S–H and relics of clinker, microcracks; (**c**) carbonated hydration products; (**d**) C–S–H and relics of clinker.

Microphotographs of the reference specimens and cement paste with addition of CFBC fly ash after 28, 200 and 400 days of maturation are presented in Figure 5 and Figure 6. It is visible as a fine-grained and fine-porous microstructure in micro-areas occupied by C–S–H, verified by EDS analysis. The C–S–H phase created a spongy mass of the conformation of small grains, generally forming single fibrils with lengths less than 0.1 μm. Ettringite needles and micro-tubes with lengths of up to 2 μm occurred in air-voids.

The addition of the CFBC fly ash, both from hard coal and lignite burning, caused an increase in the content of ettringite, assessed on the basis of the X-ray diffraction analysis. After 28 days of curing the ettringite content in the specimens with 30% T fly ash was 31% higher and with 30% K fly ash, 24% higher than in reference specimens. The content of portlandite decreased with increasing CFBC fly ash content. In specimens with 30% ash from hard coal K burning, an increase of the content of calcium carbonate over time is clearly visible. Similar observations regarding the microstructure of paste with CFBC fly ash from hard coal burning were made by Lee and Kim [37], who investigated the hydration reactivity of the CFBC fly ash. They concluded that the microstructure of pastes with CFBC fly ash (the mixing ratio of CFBC fly ash to water was set at 1.0) after 1 day of hydration consisted mainly of fibrous ettringite and various sizes of hexagonal-plate portlandite. After 91 days, the CFBC fly ash was hydrated to a considerable degree, as the reaction ratio of the anhydrous gypsum was more than 80%. The microstructure of CFBC fly ash pastes contained portlandite, ettringite, gypsum and C–S–H [37].

The phase composition of the hardened cement paste was not affected by prolonged exposure in water and temperatures of 20 ± 2 °C. Irrespective of the content of CFBC fly ash in concrete, the microstructure of the presented cement pastes is similar to the reference specimen. It can be assumed that the efficiency of mineral additives, like fluidized bed combustion fly ash in cement paste, can be similar to other non-standard fly ash (e.g., calcareous fly ash). It was found in [38] that the addition of calcareous fly ash resulted in an improvement of concrete durability. A beneficial reduction of chloride migration coefficient was observed, while the effect on the water and air permeability was similar to its effect on the compressive strength of concrete. 

## 4. Conclusions

The performed investigation revealed the following conclusions:

- Macroscopic analysis revealed differences in colour of fluidized bed combustion fly ashes, which was assumed to be correlated to carbon content. This method can be applied for preliminary evaluation of CFBC fly ash suitability as concrete additive;
- The major components in the investigated CFBC fly ash consisted of the following (in descending order of content): SiO_2,_ Al_2_O_3,_ CaO, Fe_2_O_3_ and SO_3_.
- The largest difference between analyzed fly ash was visible in CaO content, which was the result of the type of fuel. The content of the CaO in CFBC fly ash from lignite burning was two to three times higher than in fly ash from hard coal burning.
- A proper determination of the unburned carbon content in fluidized bed fly ash required separation of CaCO_3_, portlandite and non-hydrated clay minerals content from the loss on ignition data.
- The addition of CFBC fly ash for replacement of cement by 20% or 30% by weight did not induce significant changes in qualitative phase composition of hardened cement paste cured in water up to 400 days in regard to curing for 28 days.
- The addition of the CFBC fly ash resulted in increasing content of C–S–H gel and crystalline ettringite, which was indicated by the increase of water bound in hydration products and the decrease of portlandite content.
- The crystalline ettringite content in hardened cement paste containing 30% CFBC fly ash from lignite burning was higher by about 20% in comparison to cement paste without ash at 28 days of curing.

## Figures and Tables

**Table 1:** Chemical composition of circulating fluidized bed combustion (CFBC) fly ash determined by X-Ray Fluorescence Spectroscopy XRF method (wt %).

Component	CFBC Fly Ash		
	Hard Coal Burning		Lignite Burning
	S	K	T
LOI *	5.71	3.40	2.73
SiO_2_	38.80	47.18	36.47
CaO	9.80	5.84	15.95
CaO_free_ **	not tested	3.40	4.75
Fe_2_O_3_	9.59	6.80	4.40
Al_2_O_3_	23.26	25.62	28.4
MgO	2.28	0.15	1.65
Na_2_O	2.01	1.18	1.64
K_2_O	2.22	2.36	0.62
SO_3_	5.82	3.62	3.8
TiO_2_	not tested	1.08	3.84
Cl-	0.40	0.10	0.03

* Loss on ignition at 1000 °C; ** Glycol method according to PN-EN 451-1.

**Table 2:** Chemical composition and physical properties of cement, determined by XRF method (wt %).

Component	Cement CEM I 32.5 R
LOI	3.40
SiO_2_	21.40
CaO	65.06
CaO_free_ **	1.00
Fe_2_O_3_	2.53
Al_2_O_3_	4.80
MgO	1.37
Na_2_O_eq_	0.76
SO_3_	2.60
Cl-	0.07
Specific surface, cm^2^/g	3210
Density, g/cm^3^	3.09

** Glycol method according to PN-EN 451-1.

**Table 3:** Composition of pastes prepared with CEM I and CFBC (g).

Designation of Specimens	Cement	CFBC	Water
CEM I	420	0	210
S20	336	84	210
T20	336	84	210
K20	336	84	210
S30	294	126	210
T30	294	126	210
K30	294	126	210

**Table 4:** Result of macroscopic analysis of the CFBC ashes: S, T and K.

Attribute		CFBC Fly Ash		
		S	T	K
>0.045 mm, Yield (%)		22.0	36.1	19.5
Color	Whole lot	brownish-grey 5YR 4/1	light olive-grey 5Y 6	olive-grey 5Y4/1
	Fraction > 0.045 mm	light brownish-grey 5YR 6/1	light brownish-grey 5YR 6/1	brownish-grey 5
	Fraction < 0.045 mm	brownish-grey 5YR 4/1	not tested	not tested

**Table 5:** The results of compressive and flexural strength of pastes stored in water in 20 ± 2 °C after 28 days of curing (MPa).

Designation of Specimens	Flexural Strength	Comressive Strength
CEM I	4.6 ± 0.3	33.8 ± 0.6
S20	5.8 ± 0.2	47.8 ± 1.8
T20	5.2 ± 0.3	39.8 ± 2.2
K20	6.4 ± 0.4	53.3 ± 2.3
S30	5.9 ± 0.3	46.0 ± 1.4
T30	5.9 ± 0.7	35.9 ± 1.0
K30	5.9 ± 0.6	53.1 ± 2.0

**Table 6:** Selected properties of CFBC fly ash separated for the fractions below and above 0.045 mm.

Specimen	LOI *, wt %	Mass Loss at <350 °C, wt %	Relics of Coal, wt %	Portlandite, wt %	Calcium Carbonate, wt %
**S**					
Fraction > 0.045 mm	1.7	0.45	0.8	not detected	not detected
Fraction < 0.045 mm	5.8	0.74	4.4	not detected	not detected
**T**					
Fraction > 0.045 mm	3.7	0.79	0.8	not detected	4.80
Fraction < 0.045 mm	5.9	1.05	not detected	1.2	10.35
**K**					
Fraction > 0.045 mm	3.4	0.89	1.8	not detected	1.60
Fraction < 0.045 mm	5.7	0.87	4.4	not detected	1.05

* Loss on ignition at 1000 °C.

**Table 7:** XRD estimated composition of hardened cement paste after 28, 200 and 400 days of curing (CFBC T—paste with fly ash from lignite burning. CFBC K—paste with fly ash from hard coal burning. 20/30–percent content of fly ash addition).

Age, Days	Composition Parameter	Reference Paste CEM *	WZ Index for Hardened Pastes Containing CFBC Fly Ash in Relation to the Reference Paste (%)			
			T20	T30	K20	K30
28	Hydration products HI, %	17.8 = 100%	116.3	133.1	119.7	130.9
	Portlandite CH, %	16.0 = 100%	66.9	41.3	41.3	33.1
	Calcium carbonate CC, %	10.7 = 100%	100.0	91.6	112.1	103.7
	Ettringite ∑ I, a.u. **	326 = 100%	127.9	131.3	121.2	124.2
200	Hydration products HI, %	23.0 = 100%	111.7	104.3	110.0	104.3
	Portlandite CH, %	15.2 = 100%	53.9	34.9	53.9	27.0
	Calcium carbonate CC, %	13.6 = 100%	72.0	111.8	78.7	122.0
	Ettringite ∑ I, a.u. **	459 = 100%	97.8	104.1	102.8	112.9
400	Hydration products HI, %	18.0 = 100%	118.0	123.9	112.7	131.1
	Portlandite CH, %	16.4 = 100%	65.2	39.6	37.8	17.1
	Calcium carbonate CC, %	10.2 = 100%	89.2	119.6	147.1	133.3
	Ettringite ∑ I, a.u. **	380 = 100%	105.0	115.5	97.1	112.1

* The ordinary cement paste without fly ash addition after 28, 200 and 400 days is equal to 100%; ** Absolute intensity ettringite planes of symmetry (d = 9.73 i d = 5.61) in conventional units.
